# On-farm evaluation and determination of sources of variability of soybean response to *Bradyrhizobium* inoculation and phosphorus fertilizer in northern Ghana

**DOI:** 10.1016/j.agee.2018.08.007

**Published:** 2018-11-15

**Authors:** Jacob Ulzen, Robert Clement Abaidoo, Nana Ewusi-Mensah, Cargele Masso

**Affiliations:** a Department of Crop and Soil Sciences, Faculty of Agriculture, Kwame Nkrumah University of Science and Technology (KNUST), Kumasi, Ghana; b Department of Theoretical and Applied Biology, Kwame Nkrumah University of Science and Technology (KNUST), Kumasi, Ghana; c International Institute of Tropical Agriculture (IITA), Nairobi, Kenya; d International Institute of Tropical Agriculture (IITA), Ibadan, Nigeria

**Keywords:** Smallholder farmers, TSP, *Bradyrhizobium*soil type, Grain yield

## Abstract

Soybean yields on smallholder farms in sub-Sahara Africa (SSA) are far below the potential yield thus creating a huge yield gap. Interventions are thus needed to bridge this yield gap and ascertain the factors influencing the yield variation. This study evaluated the on farm response of soybean to rhizobia inoculation and or mineral P fertilizer in Northern and Upper West regions of Ghana in a single non-replicate trial using four treatments: no input (control), TSP fertilizer (P), rhizobia inoculant (I) and TSP plus inoculant (P + I). In addition, the study sought to develop a robust approach for determining responsiveness and non-responsiveness using agronomic and economic indices. The results showed that the average grain yield of plots that received P or I were higher than control plots. Higher grain yield responses were however, obtained by the plots that received combined application of P and *Bradyrhizobium* inoculant. Grain yield response in the Northern region was higher than in the Upper West region. Response to P and or I were highly variable within and between locations. The cumulative rainfall and some soil factors including soil nitrogen, phosphorus, soil type, organic carbon, pH and texture explained about 42–79% of these variations in soybean grain yield. The agronomic approach for determining responsive and non-responsiveness revealed that 17–40 % and 6–17% of the locations within the Northern and Upper West regions, respectively were responsive to P fertilization and/ or *Bradyrhizobium* inoculation. However, the economic approach indicated that 64–75% and 14–24% of the locations within the Northern and Upper West regions, respectively were responsive to P fertilization and *Bradyrhizobium* inoculation. The results imply that rhizobia inoculation is an effective strategy for increasing soybean yield and improving livelihood of smallholder farmers.

## Highlights

• Inoculation increased grain yield over the control by 24%.
• Combined application of P + I increased grain yield over the control by 37%.
• Soil and environmental factors explained up to 79% of yield variation.
• Agronomic index for responsiveness was robust than economic index.
• About 53% of the farmers that applied inoculant had VCR of ≥2.

## 1 Introduction

Soybean plays an important role in the diets of many due to its protein content. In addition, production of soybean generates income for smallholder farmers and improve their livelihood. However, its production is largely limited by the inherent low fertility nature of smallholder farms in SSA. Soybean like any other legume requires high amount of N to attain optimum growth (Hungria and Kaschuk, 2014). The low amount of soil N and P in smallholder farms, coupled with minimal or no external inputs to boost production have resulted in low grain yields. The current grain yields recorded by farmers are less than 1 t ha^−1^ and that far below the potential yield of 2.5 t ha^−1^ (Mensah, 2014; Dugje et al., 2009)

Various interventions have been proposed to address this issue but the most significant and affordable one is the provision of N and P through rhizobia inoculation and mineral P fertilization, respectively. Combined application of rhizobia inoculant and mineral P fertilizer is known to mostly increase grain yield of legumes such as soybean and cowpea. Ronner et al. (2016) reported a significant increase in grain yield of 452 kg ha^−1^ and 447 kg ha^-1^ due to rhizobia inoculation and single superphosphate application in Nigeria. Masso et al. (2016) reported a significant increase in grain yield of 426 kg ha^−1^ and 482 kg ha^-1^ due to the application of rhizobia inoculant and triple superphosphate in Ghana. Greater yield response are obtained when rhizobia inoculant and phosphorus fertilizer are combined. For instance, Ronner et al. (2016) and Masso et al. (2016) reported grain yield increases of 777 kg ha^-1^ and 631 kg ha^−1^, respectively in soybean when inoculant application was combined with phosphorus fertilizer. Kyei-Boahen et al. (2017) also reported 56% yield increase in cowpea when inoculant was applied together with P in Mozambique.

Nonetheless, soybean – rhizobia symbiosis is affected by the environment, management, rhizobia strain and legume genotype (Woomer et al., 2014). These factors determine the success or otherwise of the symbiosis in increasing yield. In situations, where only one strain of rhizobia is involved and the legume genotype is promiscuous, the environmental factors and management practices will be the major contributing factors, controlling yield. For example, Ronner et al. (2016) reported that 16–60% of the variations were explained by the environmental factors. Fermont et al. (2009); Bielders and Gerard (2015) and Falconnier et al. (2016) also reported that the environmental, management and soil factors explained 20% 58% and 49% of the variability in cassava, millet and sorghum-cowpea-soybean yields, respectively under smallholder farmer conditions. Soils in sub-Sahara Africa exhibit a wide variability in soil fertility (Giller et al., 2011) and this contributes to the limitation of the treatment potential in increasing yield and the spatial response to the treatments on smallholder farmers.

The spatial variability in soil fertility on smallholder farms in SSA has also led to the classification of soils as responsive and non-responsive (Vanlauwe et al., 2010; Kihara et al., 2016). This is of major interest and the discussion about finding appropriate method for classification is still an on going research. The current method involve setting of yield ceilings and percentages; however, this method is very subjective. Kihara et al. (2016) used K-means clustering to determine maize response to fertilizer in their nutrient omission trial setting a yield threshold of 3 t ha^−1^.

The N2Africa and COMPRO II Projects have disseminated legume rhizobia technology to smallholder farmers in the northern Ghana aiming at high adoption rates by the farmers. Given that adoption of such technologies represent risk of forgoing their current practices, it is imperative to establish which locations within the region will demonstrate effective and stable crop responses. Although, it is true that the spatial variation in nutrients on smallholder farms causes yield variation, little is known of the magnitude and direction (positive or negative) of such effects. This study therefore sought to (i) evaluate the on-farm response of rhizobia inoculant and or mineral P fertilizer; (ii) develop a robust approach for determining responsive and non-responsiveness using agronomic and economic indices; and (iii) identify the major factors limiting soybean response on smallholder farms in northern Ghana. This work will allow for better targeting of future dissemination technologies to areas where the potential of the treatments could be maximized. In addition, having knowledge of the factors limiting soybean response to inoculation and phosphorus application will lead to initiation of measures to address these challenges.

## 2 Materials and methods

### 2.1 Study area

Agronomic trials for testing the response of soybean to rhizobium inoculant and phosphorus fertilizer were set up in Northern region (Savelugu – Nanton and Gushiegu - Karaga districts) and Upper West region (Sissala West, Sissala East and Wa municipal) during the 2015 cropping season as illustrated in Figs. S1 and S2. The rainfall pattern in the study locations is unimodal with an average annual rainfall of 1000–1200 mm and mean temperature between 26 and 30 °C with little variation throughout the year. The rainfall data were downloaded from www.awhere.com

### 2.2 Soil sampling and analyses

Seven soil core samples were taken from each plot, thoroughly mixed and composite samples taken into transparent polythene bags and kept in a refrigerator at 4 °C prior to laboratory analysis. The soil parameters analysed were particle size (hydrometer method), soil pH (1:2.5) (H_2_O), organic carbon (modified Walkley and Black procedure as described by Nelson and Somers (1996), total nitrogen (Kjeldahl method as described by Bremner and Mulvancy (1982), available soil phosphorus (Bray No. 1 solution as outlined by Olsen and Sommers (1982) and exchangeable potassium (ammonium acetate (NH4OAc) extract. Calcium and magnesium were determined in 1.0 *M* ammonium acetate (NH4OAc) extract (Black, 1965). Active carbon was determined following the procedure of Culman et al. (2012).

### 2.3 Training of agricultural extension agents (AEAs) on protocol (treatments)

Due to the large number of demonstration sites, the experiment was conducted in partnership with AEAs and farmers. It was imperative to equip the AEAs with technical knowledge for successful implementation of the trials. The training focused on the handling, application of rhizobium inoculant and phosphorus fertilizer, selection of sites, good agronomic practices and data collection.

### 2.4 Mobilization of farmers

Northern and Upper West regions were selected for the study due to the predominance of soybean cultivation in those two regions. Farmers in the selected locations within each district had been previously introduced to legume-inoculant technology by non-governmental organizations and therefore understood the demands of the technology. Mobilization of farmers was done through community sensitization and education about improved soybean technologies with the AEAs. Interested farmers were selected by the AEAs, organized into groups of 20–25 people. Within farmer groups, lead farmers were selected and trained on the handling and application of *Bradyrhizobium* inoculant, phosphorus fertilizer application and good agronomic practices. Each farmer received an improved soybean variety, rhizobium inoculant (Nodumax) and triple super phosphate (TSP). As a requirement, farmers were asked to set up the trials at locations visible to others especially non-participating farmers.

### 2.5 Field preparation, layout, inoculation and sowing

Each field was ploughed and harrowed to a depth of 15 cm and divided into 4 plots measuring 10 m x 10 m with an alley of 1 m. The soybean seeds were sown at a distance of 75 cm x 10 cm. The soybean cultivar, Jenguma (TGx series) was used. Five grams of the *Bradyrhizobium* inoculant was added to 1 kg of seeds and applied using the two-step method (Somasegaran and Hoben, 2012). Planting between the districts were done in a week interval and within a week for each district with the help of AEAs. In the Northern region, planting was done between 7 – 13th July 2015. In the Upper West region, planting was done between 15–21 August 2015.

### 2.6 Treatments and experimental design

There were four (4) treatments: inoculant only (I), TSP (only) (P), no input (control) and a combination of TSP and inoculant (P + I). The treatments were tested in a simply non- replicated trial where each farm within a district was considered a replicate. The rhizobium inoculant (Nodumax) contained 10^9^ cells g^−1^ of *Bradyrhizobium japonicum* strain USDA 532c. The TSP (46% P_2_0_5_) was applied at a rate of 30 kg P ha^−1^. The mode of application was band placement. About 136 and 45 demonstration trials were established in the Northern and Upper West regions, respectively with the help of farmers and AEAs.

### 2.7 Data collection

Soils were sampled for physical and chemical analyses as well as enumeration of indigenous rhizobia population before planting. Rhizobia population was assessed using the most probable number technique. At maturity, the soybean plants were harvested, threshed and winnowed. The seeds were air dried until constant weight was attained and weighted accordingly with standard electronic scale. Grain yield was estimated on per hectare basis.

### 2.8 Determination of responsive and non-responsive sites

For the purpose of this work, responsiveness and non - responsiveness were defined by agronomic and economic indices. For the agronomic index, the average of the total yields of the control from the different locations were calculated. Standard deviation was calculated from this average and used as a threshold for comparison. Differences between treatment and control yields were compared to the standard deviation; where differences were higher than the standard deviation, the location was considered responsive, and non-responsive, when where differences were lower than the standard deviation. The rationale is that the standard deviation was a representative of all the locations under consideration. Differences less than the standard deviation was considered as a random variation in the population while differences higher than the standard deviation was attributed to the effect of the treatments. The computer software ArcGIS was used to map out the responsive and non-responsive soil locations.

The economic index used value cost ratio as estimation option. The ability to recover (break –even) or make profit after application of fertilizer at a particular location was considered as responsive and vice versa. The rationale is that if a particular soil is not good productively, then the cost of fertilizer cannot be recovered after application due to low grain yield. The market price of USD$ 0.43 per 1 kg soybean seeds was used (GH 1.5 at an exchange rate of GH 3.5 to 1 USD). *Bradyrhizobium* inoculant and triple superphosphate (TSP) were procured at the cost of 6 US$ ha^−^ and 26 US$ ha^-1^, respectively. Labour for planting and application of fertilizer was estimated at 17 US$ ha^-1^.

### 2.9 Statistical analysis

Absolute ad relative responses of soybean to P and / or inoculant in relation to the control of the individual locations were calculated based on the formula of Ronner et al. (2016) and expressed as cumulative probability curves. Statistical analyses were performed in R version 3.3.2 (R Core Team, 2017). The effects of the treatments were estimated with linear mixed model: treatment as fixed term and location as random term. Treatment means were separated by lsmeans with Tukey adjusted p-values. Linear mixed model regression was performed to identify the soil and environmental factors influencing yield variability. Only locations with complete data set were used in the analysis.

## 3 Results

### 3.1 Soil chemical and physical properties

The ratings for the soil chemical and physical properties were done according to the classification by Landon (2014). In the Northern region, organic carbon values recorded were very low (Table 1). Similarly, available P was low with little variation across the different locations. The total nitrogen contents of the study sites were largely very low. The total N concentration values ranged from 0.03 – 0.13% across locations in Northern region. Thirty three percent, (33%) of the study locations in the Northern region had low nitrogen content and the remaining had very low nitrogen content. The exchangeable potassium was also very low. The values for exchangeable calcium ranged from low (2.8 cmol _(+)_ kg^−1^) to medium (11.44 cmol _(+)_ kg^−1^) with much variation between some of the locations. The values obtained for exchangeable magnesium were between medium (0.30 cmol _(+)_ kg^−1^) and very high (4.06 cmol _(+)_ kg^−1^) with much variation between locations. The locations had soils with relatively large amounts of silt and low amounts of sand and clay. The pH ranged from medium (5.60) to high (6.99) (Table 1).

**Table 1:** Soil physical and chemical properties of study locations.

Northern Region (N = 85)			
Soil parameters	Median	Minimum	Maximum
pH(1:2.5)	6.19	5.60	6.99
Total N (%)	0.084	0.031	0.125
Available P (mg kg^−1^)	5.69	5.200	12.70
Exchangeable K (cmol _(+)_ kg^−1^)	0.02	0.009	0.047
Organic C (%)	0.86	0.320	1.520
Exchangeable Ca (cmol _(+)_ kg^−1^)	4.72	2.080	11.44
Exchangeable Mg (cmol_(+)_ kg^−1^)	1.86	0.300	4.06
Sand (%)	71.96	45.04	86.08
Clay (%)	6.88	2.960	10.52
Silt (%)	21.64	5.40	50.0

Upper West Region (N = 20)			
pH(1:2.5) (H_2_O)	6.34	5.64	7.56
Total N (%)	0.058	0.038	0.11
Available P (mg kg^−1^)	7.09	6.040	9.90
Exchangeable K (cmol _(+)_ kg^−1^)	0.012	0.0050	0.029
Organic C (%)	0.64	0.40	1.22
Exchangeable Ca (cmol _(+)_ kg^−1^)	2.51	1.62	5.66
Exchangeable Mg (cmol_(+)_ kg^−1^)	0.76	0.16	2.24
Sand (%)	76.02	47.64	87.6
Silt (%)	19.48	9.28	47.28
Clay (%)	4.72	3.080	8.360

In the Upper West region, there was little variation between locations regarding soil organic carbon contents (Table 1). The organic carbon was very low across locations with a median of 0.64%. Available phosphorus ranged from low to medium. Only 5% of the 20 locations had low nitrogen content with the remaining locations having very low (0.038%) nitrogen. Exchangeable magnesium was generally high in 50% of the locations Thirty percent (30%) of the locations had medium exchangeable magnesium and the remaining 20% had low amount of exchangeable magnesium. Majority of the locations had low exchangeable calcium while about 25% of the locations had medium amount of exchangeable calcium. The soils had relatively high sand (76%) and low clay content (4.7%). There was virtually no variation in exchangeable potassium between the sites and were described as very low. The pH ranged from medium (5.64) to high (7.56) (Table 1).

### 3.2 Indigenous rhizobia population in the study locations

Considerable variation existed between locations in each region and between regions in indigenous rhizobia populations. The population sizes were relatively higher in soils of Northern region than soils in Upper West region (Table 2).

**Table 2:** Indigenous rhizobia population (cells g^−1^ soil) of the study locations.

Location	Median	Minimum	Maximum
Northern region (N = 69)	57.1	11.4	1464.9
Upper West region (N = 20)	91.7	1.1	287.1

There were significant differences among the indigenous rhizobia populations across the various locations in the Northern region. The population ranged from as low as 11.4 to 1464 rhizobia cells g^−1^ soil. More than 50% of the locations had rhizobia numbers less than 100 cells g ^−1^ soil. Within the 50%, more than half-recorded numbers less than 50 cells g^−1^ soil. The median rhizobia population in the soils of Northern region was 57.1 cells g^−1^ soil (Table 2).

Similarly, in the Upper West region, there was significant variation among the indigenous rhizobia population sizes between the locations. The highest indigenous population recorded was 287 cells g^−1^ soil and the least was 1.1 cells g^-1^ soil. The indigenous rhizobia population sizes of 50% of the locations were above 100 cells g^-1^ soil and 45% had indigenous population of less than 50 cells g^-1^ soil. The median rhizobia population in the Upper West region soils was 91.7 cells g^-1^ soil (Table 2).

### 3.3 Rainfall

In the Upper West region, there was rainfall after planting until day 30. Thereafter, the rainfall seldom reached 20 mm per day, culminating into dry spells just before and after flowering. In addition, there were short dry spells after flowering that continued until harvesting (Fig. S3).

In the Northern region, there was a dry spell after the day 10 up to day 40 after planting. Thereafter, there was adequate rainfall until flowering with short dry spells up to podding. The total rainfall received at the Northern region was higher than that of Upper West region (Fig. S4).

### 3.4 Soybean grain yields

The average grain yield from plots that received P and / or inoculant (I) were significantly (p < 0.0001) higher than those of the control plots at the study locations in Northern region (Table 3). Phosphorus and inoculant effects resulted in 18% and 24% increase in grain yield over the control, respectively in the Northern region. The P + I treatment recorded the highest grain yield of 1371 kg ha^−1^ (Table 3).

**Table 3:** Average soybean grain yields in Northern and Upper West regions.

Treatment	Northern region	Upper West region
	kg ha^−1^	
Control	998.41 ± 44.6* c^†^	213.04 ± 15.8 b*†
TSP (P)	1177.00 ± 8.3 b	263.53 ± 9.3 a
Inoculant (I)	1237.52 ± 2.9 ab	236.67 ± 5.3 ab
TSP plus inoculant (P + I)	1370.75 ± 9.7 a	271.86 ± 4.8 a
*P-value*	< 0.0001	0.0003

^†^Within column, means followed by same letters are not different at 0.05 probability level.

^*^Standard error of the mean.

Unlike, the Northern region locations, the grain yields recorded at the study locations in the Upper West region study were low; variations between locations were also significant (p = 0.0003) (Table 3). There were significant differences between control plots and plots that received P only, P + I but not with plots that received inoculant (I) only. Plots that received I + P also produced the highest grain yield in Upper West region (Table 3).

### 3.5 Distribution of soybean responses to TSP and Bradyrhizobium inoculation in the northern region of Ghana

**Fig. 1:**
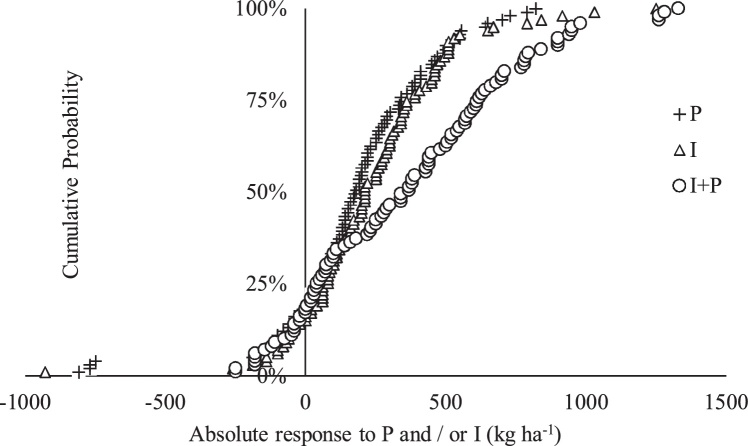
Cumulative probability of estimated absolute response of soybean grain yield in the Northern region.

In absolute terms, 81, 83 and 81% of the locations had a positive response to P, inoculant (I) and P + I, respectively, in relation to the control in the Northern region (Fig. 1). Forty four percent of the farmers increased their grain yields in absolute terms by about 200 kg ha^−1^ or more with phosphorus only.

**Fig. 2:**
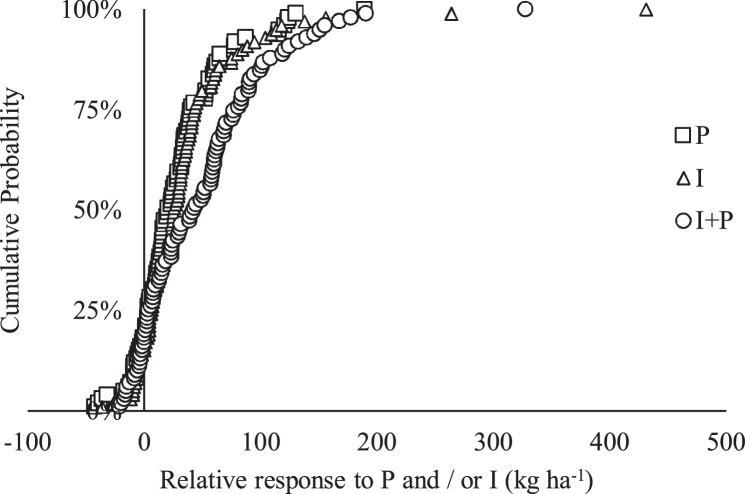
Cumulative probability of estimated relative response of soybean grain yield in the Northern region.

About 56% of the farmers increased their grain yields by at least 200 kg ha^−1^ with inoculant only. Sixty-two percent of the farmers had absolute increase in grain yield of at least 220 kg ha^−1^ with inoculant and phosphorus combined (I + P). Gains of 1000 kg ha^−1^ grain yield or more were achieved by 2% of the locations where the plots were inoculated only and 4% with locations that received P + I. None of the locations that received P only had yield gains of 1000 kg ha^−1^ or more (Fig. 1). The probability of achieving a negative response due to the application of P, inoculant (I) and / or P + I were 18, 14 and 16%, respectively (Fig. 2).

More than half of the locations recorded relative grain yield of 20% or more with P, 20% or more with inoculant and 23% or more with P + I. Seven percent of the locations achieved over 100% relative increase in grain yield with P, 8% with inoculant (I) and 15% with P + I (Fig. 2).

### 3.6 Distribution of soybean responses to TSP and Bradyrhizobium inoculation at Upper West Region of Ghana

**Fig. 3:**
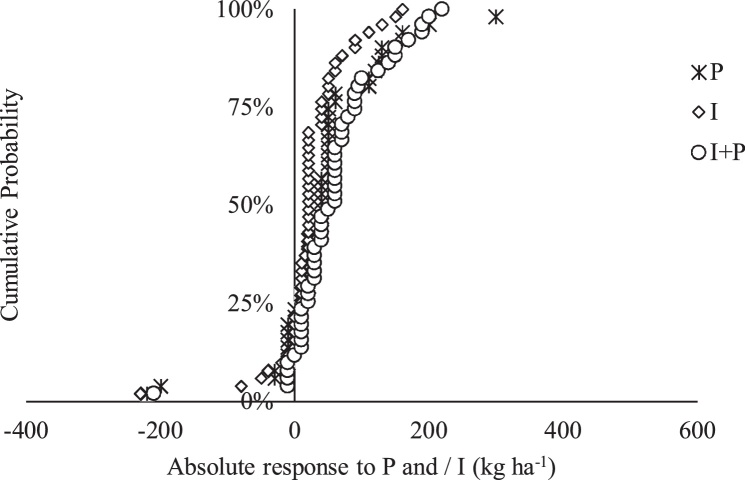
Cumulative probability of estimated absolute response of soybean grain yield in the Upper West region.

In absolute terms, 75, 76 and 86% of the locations had a positive response to P, inoculant (I) and P + I, respectively, relative to the control in the Upper West region (Fig. 3). Gains of at least 100 kg ha^−1^ was obtained from 22% of the locations that received P, 8% that received inoculant and 18% that received P + I. None of the locations had yield gain of 1000 kg ha^−1^ (Fig. 3). The probability of achieving a negative response due to the application of P, inoculant and P + I were 20, 12 and 10%, respectively (Fig. 3).

**Fig. 4:**
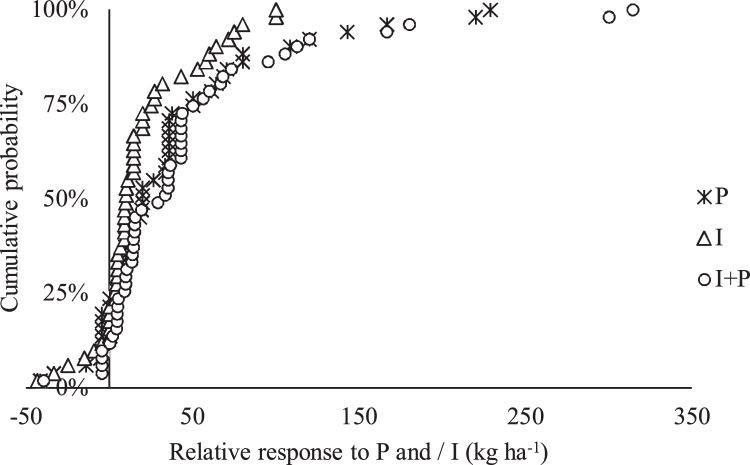
Cumulative probability of estimated relative response of soybean grain yield in the Upper West.

Half of the farmers increased their grain yield by 20% or more with P, 10% or more with inoculant use and 29% or more with P + I (Fig. 4). On 4, 12 and 14% of the locations, relative increase in yield of 100% or more was achieved with inoculant use, P and P + I, respectively (Fig. 4).

### 3.7 Variability in soybean grain yield and response to P and / or I

**Fig. 5:**
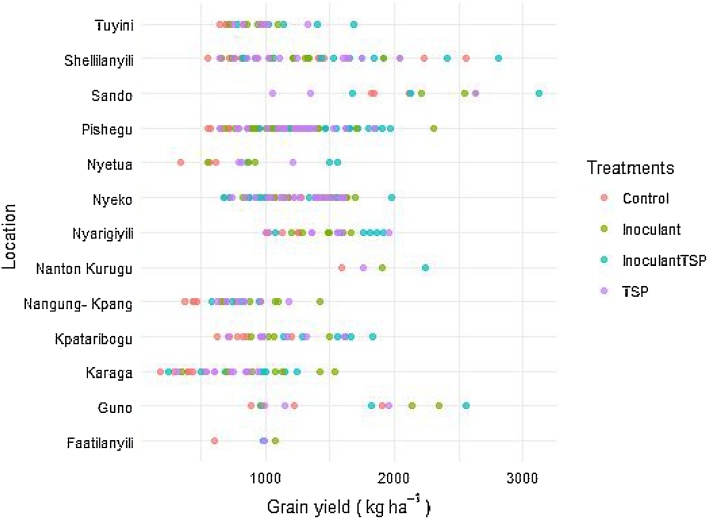
Variability in grain yield response to TSP and / or Inoculants in the Northern region.

**Fig. 6:**
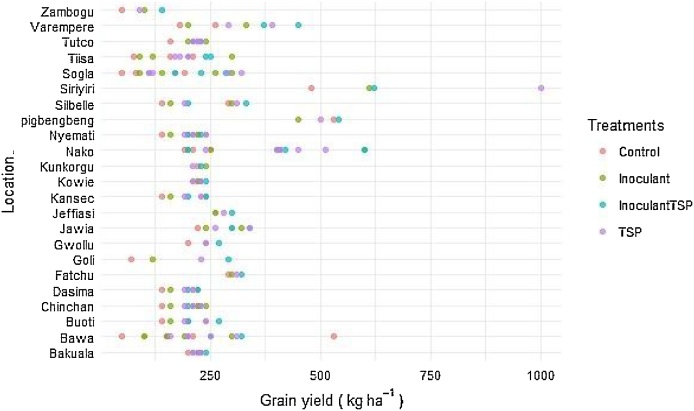
Variability in grain yield and response to TSP and / or Inoculant in the Upper West region.

Fig. 9, Fig. 10 show the performance of the treatments at the various locations. There was a wide variation in grain yield among the treatments and between locations (Fig. 5, Fig. 6). Grain yields on the control plots ranged from 180 to 2560 kg ha^−1^ while those of the treated plots ranged from 250 to 3120 kg ha^−1^ for Northern region (Fig. 5). Except at Sheillianyilli, grain yields for all control plots at the various locations were below 2000 kg ha^−1^ (Fig. 5).

Grain yields in control plots ranged from 50 to 600 kg ha^−1^ while those of the treatments ranged from 90 to 1000 kg ha^−1^ for Upper West region (Fig. 6). The least grain yield was recorded at Bawa with the control treatment while the highest yield was recorded at Siriyiri with phosphorus application (Fig. 6).

### 3.8 Economic viability of using P and / or I in the Northern Region and Upper West regions

**Fig. 7:**
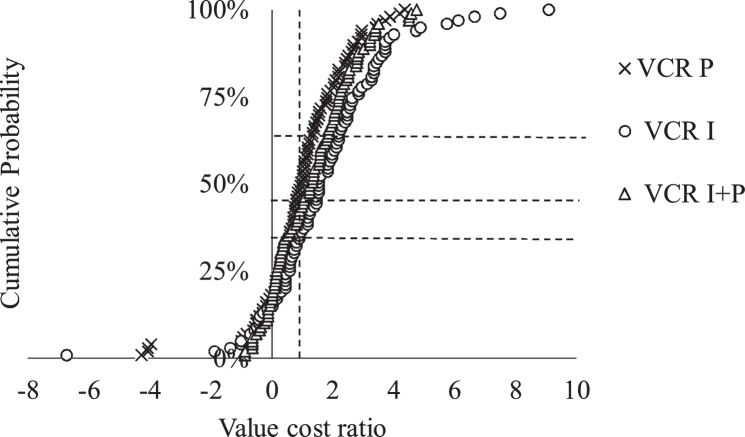
Cumulated probability of estimated value cost ratio of P and / or I in the Northern region. The cumulative probability (Y-axis) reflects the likelihood for obtaining a value larger than a given VCR (X-axis). Vertical line denotes VCR = 1 and horizontal lines intersect with the cumulative distribution curves for I, P and P + I in that order.

**Fig. 8:**
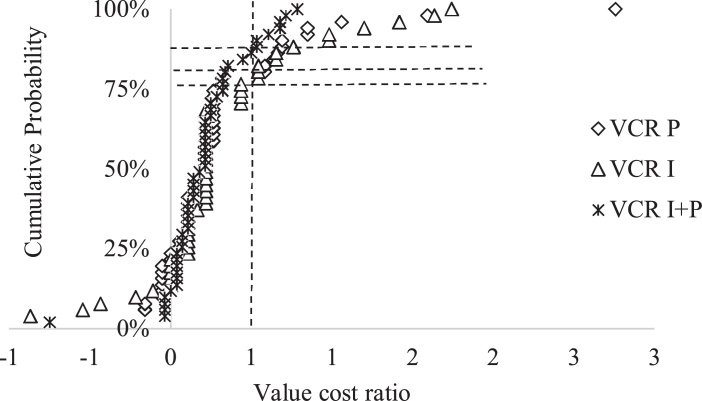
Cumulated probability of estimated value cost ratio of P and / or I in the Upper West region. The cumulative probability (Y-axis) reflects the likelihood for obtaining a value larger than a given VCR (X-axis). Vertical line denotes VCR = 1 and horizontal lines intersect with the cumulative distribution curves for I, P and P + I in that order.

The probability of achieving economic benefit which reflects on the responsiveness and non-responsiveness to P and or inoculant (I) compared to the control is presented as probability distribution graph (Fig. 7, Fig. 8). Fig. 7 shows that the inoculation treatment values are more shifted to the far right than P and P + I indicating that the use of inoculant would be more profitable. About 66% of the farmers who applied P had gross returns equal to or greater than the cost of applying P. Out of the 66% farmers, 35% had VCR of 1, 18% had VCR of 2, 9% had VCR of 3 and 4% had VCR of 4 (Fig. 7). For inoculant application, 22% of the farmers had VCR of 1, 24% had VCR of 2, 15% had VCR of 3 and 14% had VCR ranging from 4 to 9 (Fig. 7). For P + I, the ratios were much less for farmers that had VCR of 1 than that of P and I only. However, 19% of the farmers who applied P + I had VCR of I, 27% had VCR of 2, 14% had VCR of 3 and 4% had VCR in the range of 4–5 (Fig. 7).

**Fig. 9:**
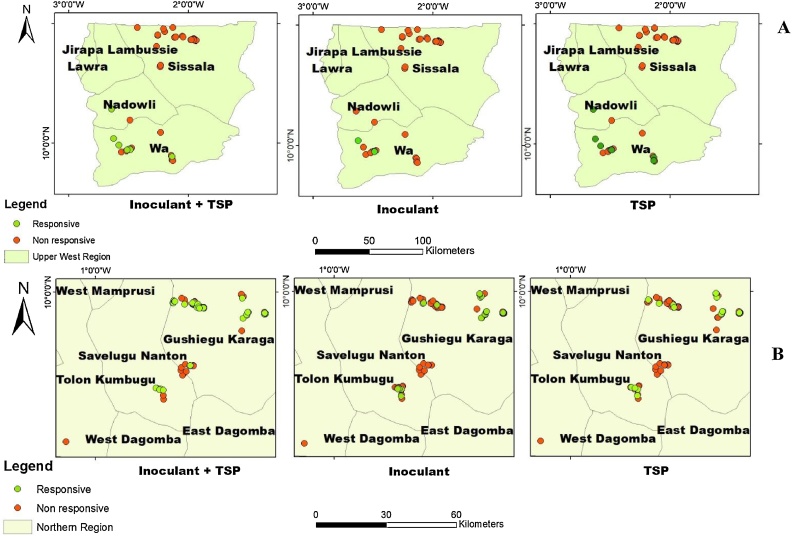
Soybean response to P and / or inoculant in the Northern (A) and Upper West (B) regions.

**Fig. 10:**
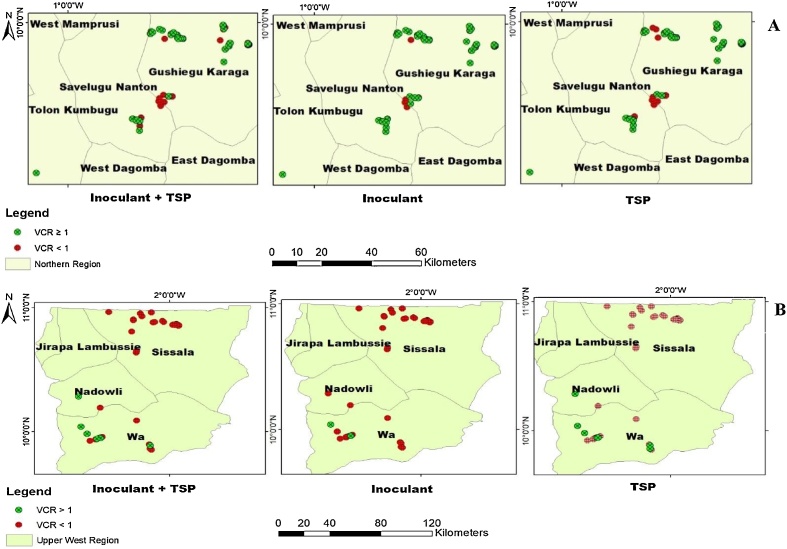
Value cost ratio for phosphorus and / or inoculant use in the Northern (A) and Upper West (B) regions.

A large proportion of farmers in the Upper West region recorded a VCR of zero (Fig. 8). Twenty - two percent of them who applied P had a VCR of one or more. Out of the 22%, only two percent had a VCR of 2 and 3 (Fig. 8).

Twenty-four percent of the farmers who used inoculant had a VCR of one or more. Out of the 24%, four percent had a VCR of two (Fig. 8). Fourteen percent of the farmers who applied the inoculant with phosphorus had a VCR of one. None of the farmers who applied the inoculant with phosphorus had a VCR of two or more (Fig. 8). The VCRs for inoculant and phosphorus are more shifted towards the right than P + I indicating that inoculant and phosphorus use were more profitable in Upper West region.

### 3.9 Responsive and non – responsive sites to P and inoculant application to soybean

There was wide variation in soybean response to P and / or I. Based on the agronomic approach described under Section 2.8, seventeen percent (17%) of the sites within Northern region were responsive to P, 21% responsive to inoculant and 40% responsive to P + I (Fig. 9 A). Majority of the trial sites were either non – responsive to P and or inoculant (Fig. 9A). Seventeen (17%) percent of the sites within Upper West region were responsive to P and P + I (Fig. 9B). Only 6% were responsive to inoculant (Fig. 9B).

If we consider, the economic approach, the picture changes as many sites become responsive. About 66% of the sites within Northern region were responsive to P, 75% responsive to inoculant and 64% to P + I (Fig. 10 A). Only 22% of the sites within Upper West region were responsive to P, 24% to inoculant and 14% to P + I (Fig. 10B).

### 3.10 Understanding the variability in soybean yield response to P and / or I

Overall, the linear model explained 42% of the total variances in grain yield in the Northern region (Table 4). Soil factors such as nitrogen and phosphorus had positive significant effect on soybean grain yield. Cumulative rainfall and soil types had significant negative effect on grain yield. Native rhizobia population had negative effect on grain yield, though it was not significant (Table 4).

**Table 4:** Explanatory variables for variability in grain yield in selected locations of the Northern region.

Coefficients	Estimate	standard error	t value	Pr (>|t|)
(Intercept)	1.94E+05	6.93E+04	2.796	0.0059 **
Nitrogen	1.04E+03	2.86E+02	3.627	0.0004 ***
Organic carbon	3.50E+02	1.80E+02	1.95	0.05
Phosphorus	2.21E+02	1.09E+02	2.024	0.045 *
Potassium	−2.19E+04	6.78E+03	−3.226	0.0015 **
Calcium	−2.68E+00	2.13E+01	−0.126	0.90
Magnesium	−1.02E+02	5.63E+01	−1.81	0.07
Cumulative rainfall	−3.19E+02	1.13E+02	−2.809	0.0056 **
Native rhizobia	−1.18E-01	2.87E-01	−0.412	0.68
Active carbon	2.60E-01	2.02E-01	1.287	0.20
pH	1.97E+02	1.42E+02	1.391	0.17
% Sand	−2.62E+00	5.83E+00	−0.448	0.65
% Clay	1.11E+01	2.49E+01	0.444	0.66
Texture_silt	−1.20E+02	3.09E+02	−0.388	0.70
Texture_silt loam	1.10E+02	2.61E+02	0.42	0.68
Soil_type_Dysteric Plinthosols	−5.68E+02	9.13E+01	−6.22	5.04e-09 ***
Soil type_Ferric Lixisols	−6.26E+01	1.52E+02	−0.413	0.68
Soil type_Planosols	2.22E+02	1.48E+02	1.497	0.14
Soil type_Pinthic Lixisols	−8.80E+02	1.92E+02	−4.578	1.00e-05 ***
Adjusted R-squared : 0.42				
F-statistic: 7.461 on 18 and 145 DF, P-value: <0.0001				

Significant levels: *p < 0.05, **p < 0.01, and ***p < 0.001.

On the contrary, the linear model explained 79% of the variance in grain yield in the Upper West region (Table 5). Soil nitrogen and organic carbon had negative effect on grain yield. The effect of phosphorus and pH were significantly positive. Unlike the Northern region, soil types had positive significant effect on grain yield. Again, native rhizobia had negative effect on grain yield.

**Table 5:** Explanatory variables for variability in grain yield in selected locations of the Upper West region.

Coefficients	Estimate	standard error	t value	Pr (>|t|)
(Intercept)	2.78E+03	9.99E+02	2.79	0.008 **
Nitrogen	−3.16E+03	1.28E+03	−2.463	0.0177 *
Organic carbon	−6.77E+02	2.68E+02	−2.521	0.015 *
Phosphorus	1.23E+02	5.17E+01	2.389	0.021 *
Potassium	5.75E+03	3.51E+03	1.639	1.08E-01
Calcium	3.28E+01	3.72E+01	0.881	3.83E-01
Magnesium	−1.49E+02	9.42E+01	−1.578	1.21E-01
Native rhizobia	−5.53E-01	4.26E-01	−1.298	2.01E-01
Active carbon	−0.06916	1.39E-01	−0.498	0.62
pH	1.17E+02	4.40E+01	2.66E+00	0.011 *
% Sand	−5.77E+01	1.86E+01	−3.099	0.003 **
% Clay	5.38E+01	2.54E+01	2.118	0.040 *
Texture_sandy loam	−1.03E+03	3.50E+02	−2.948	0.0051 **
Soil type_Ferric Lixisols	7.61E+02	3.93E+02	1.936	0.06
Soil type_Leptosols	7.71E+02	3.36E+02	2.296	0.026 *
Adjusted R-squared : 0.79				
F-statistic: 17.68 on 14 and 45DF, *P-value:* <0.0001				

Significant levels: *p < 0.05, **p < 0.01, and ***p < 0.001.

## 4 Discussion

### 4.1 Soybean response to TSP fertilizer and Bradyrhizobium inoculation

Soybean responded significantly to *Bradyrhizobium* inoculation and phosphate fertilizer application in the Northern region. The average yields obtained in this study were within the range reported by Masso et al. (2016) who conducted similar research activity in 2014 at Savelugu – Nanton and Karaga district and Ronner et al. (2016) in Nigeria. However, it was in contrast with the findings of Falconnier et al. (2016) who did not observe a significant increase in soybean grain yield after applying *Bradyrhizobium* inoculant. The difference in the two results could be attributed to a number of factors including the quality of the inoculant, the initial soil nitrogen concentration and native rhizobia populations. Falconnier et al. (2016) reported a range of 0.28 – 0.33% of soil N which was 3–9 times higher than the range 0.03 – 0.13 obtained in this work. Higher nitrogen tend to limit the activities of introduced rhizobia. Given that the soils in the Northern region had very low N and P, it was not surprising that external inputs like *Bradyrhizobium* inoculant and P significantly increased grain yield. The *Bradyrhizobium* inoculant enhanced the plants access to nitrogen through biological nitrogen fixation (Masso et al., 2016). Likewise, the phosphate fertilizer enhanced access to P. Apart from P supplying ATP, which is the energy needed for symbiosis and for the overall growth of the host legume, P is involved in every single activity leading to nitrogen fixation (Keyser and Li, 1992; Crews, 1993; O’Hara, 2001). When P and inoculant were applied together, greater response was obtained which confirms the significance of P nutrition to legume-rhizobia symbiosis (O’Hara, 2001). The responses to inoculation obtained in the Upper West region, however, were not significant. This observation is in tandem with the reports by Okogun and Sanginga (2003) and Falconnier et al. (2016). The median native rhizobia population for the Upper West region was 91 cells g^−1^ soil, which could have obviated significant response to inoculation. Response to rhizobia inoculation is not likely when native rhizobia population is above 10 (Sanginga et al., 1996; Houngnandan et al., 2000) and up to 50 cells g^−1^ soil (Slattery et al., 2004). In general, the yields obtained in the Upper West region were very low which could be attributed to the poor rainfall received during the cropping season (Fig. 3, Table 3).

### 4.2 Variability in soybean grain yield

There was variation in grain yield between locations and among treatments due to the spatial variability in soil nutrients and environmental factors. This seems to be a common characteristics of on – farm trials in smallholder settings in SSA as reported by several other researchers (e.g., Zingore et al., 2007; Fermont et al., 2009; Bielders and Gérard, 2015; Diarisso et al., 2016; Falconnier et al., 2016; Kihara et al., 2016; Masso et al., 2016; Ronner et al., 2016). The variables measured in this experiment could explain 42–79% of the variances in grain yield in the Northern and Upper West regions. This finding is comparable to that of Ronner et al. (2016) who found out that soil, environmental and management factors explained 16–61% of the variability in soybean grain yields under similar experimental conditions in Nigeria. Fermont et al. (2009); Bielders and Gérard (2015) and Falconnier et al. (2016) also reported that environmental, management and soil factors explained 20, 58 and 49% of the variability in yields of cassava, millet and sorghum – cowpea - soybean sole or intercropping systems, respectively under smallholder farmers conditions. The fact that the soil and environmental factors measured could explain 42% of the variability in Northern region indicates that other factors that were not identified by this study might have also contributed to the yield variability. It is typical of on farm trial that large proportions of the variability remain unexplained (Bielders and Gérard, 2015; Falconnier et al., 2016) but treatment contributions to the yield variability cannot also be over looked. Bielders and Gérard (2015) reported that the applied treatments contributed to 27% of the variation in the millet grain yield. Soil constraints are not the only driving forces for productivity; management decisions by farmers do affect productivity too (Dang and Moody, 2016). The study did not consider management as a variable factor as it has been the case of other studies. This is because we trained and employed Agricultural Extension Agents (AEAs) who ensured that farm activities were standardized across and therefore could contribute little to the variation in grain yield.

Soil nitrogen, active and organic carbon had positive effect on yield in the Northern region. Though the current level of these nutrients are low, recording positive effect indicates that plant growth were not limited by these nutrients, which might have rather promoted responses to inoculation. In contrast, these factors had negative effect on the yield in the Upper West region indicating that plant growth was limited by the current levels of the nutrients. Sorption of P is likely to be the major reason for non-responsiveness to P in many of the locations despite the initial low levels of P in the soil. Potassium had negative effect on grain yield indicating the low levels of potassium at the study locations were limiting the effect of the treatments. In a nutrient omission trial, potassium omission resulted in yield reduction in maize and soybean (Seitz, 2014; Kihara et al., 2016). Magnesium content was high and had negative effect indicating that such levels were not desirable for the applied treatments. Considering rhizobia inoculation, it can be argued that soil with good fertility can provide nutrients for plant and rhizobia to ensure effective symbiosis. On the contrary, soils with poor fertility do hinder effective symbiosis due to poor crop nutrition. Percent sand had negative effect on yield whereas percent clay had positive effect on yield. Sand is known to have poor water holding capacity and does not support rhizobia survival (Zengeni et al., 2006). Furthermore, leaching of nutrients are high with sandy soils. Therefore, its negative effect on yield was not surprising. Soil texture, soil type, Mg, pH, N and rainfall have all been reported to influence yield variability in smallholders’ farm (Fermont et al., 2009; Falconnier et al., 2016; Ronner et al., 2016). The native rhizobia population had negative effect on grain yield. This confirms the assertion by Thies et al. (1991) that grain yield of legumes are inversely related to native rhizobia population. At locations (e.g. Nyeko and Sheillianyilli) where native rhizobia populations were relatively high, responses to inoculation were low. Falconnier et al. (2016) reported similar observations under smallholder farm condition in Mali. Soil type influences soil nutrients that acts as covariates consequently determining crop response (Falconnier et al., 2016). Coincidently, locations (e.g., Nyeko and Sheillianyilli) in the Northern region with soil types such as Dysteric Plinthosols, and Plinthic Lixisols, respectively, were largely non-responsive to the applied treatments. Generally, Plinthosols are inherently poor in fertility due to strong weathering with underlying hardpan, which limits rooting volume and penetration (IUSS, 2014) and affects nutrient uptake and distribution. Lixisols on the other hand also have low plant nutrients and low clay activity (IUSS, 2014). Even though, the initial N and P on these soils were low, the N and P supplied through Bradyrhizobium inoculation and phosphorus fertilization could not elicit significant response. It therefore, indicates that other nutrients in limiting quantities were controlling yield. Cumulative rainfall had negative effect on yield, which is comparable to the observations by Ronner et al. (2016) in Nigeria. Diarisso et al. (2016) attributed yield variability in crops in Burkina Faso to rainfall. The negative effect of cumulative rainfall on soybean grain yield in Northern region is difficult to explain. However, two possible scenarios may be considered; excessive rainfall is likely to cause leaching or waterlogging or increase the incidence of fungal disease, which eventually affect yield. The other scenario is the shortage of rainfall, which affects nutrient uptake and limit the ability of rhizobia to fix nitrogen. The latter may partly explain the observation of this research because there were short dry spell after flowering. Rainfall was expected to be the dominant factor explaining the variability in yield at the study locations in the Upper West region because of the low rains received especially during and after flowering but this was not the case. Late planting due to late rains could be a major contributory factor for the very low yields recorded in the Upper West region. Many researchers notably Bielders and Gérard (2015) and Fermont et al. (2009) have also attributed low yields to late planting.

The agronomic approach adopted for determining responsive and non-responsive sites indicated that a large majority of the fields were non-responsive to P and / or I. Only 17–40% of the study fields in the Northern region were responsive while 6–17 % were responsive in the Upper West region. The agronomic approach has less sites being responsive in comparison to the economic approach; this shows the robustness and conservative nature of the agronomic approach. This result is comparable to Kihara et al. (2016) who reported that 11 and 25% of fields sown with maize were responsive and non-responsive to fertilizer respectively. Kihara et al. (2016) used K-means clustering to determine maize response to fertilizer in their nutrient omission trial setting a yield threshold of 3 t ha^−1^. The idea of setting threshold including certain percentage yield increase to determine responsiveness and non-responsiveness is very subjective and can lead to either over estimation or under estimation. If non-responsiveness is caused by other factors such as seasonal rainfall or management practices, other than inherent properties of the soil, it could easily be addressed. It is worth noting that though farmers do not benefit from substantial yield increases, they however, benefit from improvement in soil fertility when they incorporate the crop residues for subsequent cropping. Crop residues have been reported to contribute to soil organic matter pool (Nezomba et al., 2015). The mean grain yields showed significant responses to the applied treatments in general but it did not provide clearer information on the treatment performance of the individual farms. The cumulative probability curves showed the performance of the treatments on individual farms and therefore indicated what will happen should farmers forgo their practices and adopt these treatments (Vanlauwe et al., 2016). Therefore, it will be misleading to make general recommendations for all farmers based on the averages (Bielders and Gérard, 2015; Ronner et al., 2016). Recommendations should be based on individual farm performances and risks associated with the adoption in terms of economic benefits spelt out to farmers.

### 4.3 Economic viability of P and/ or I

Value cost ratio (VCR) is a simple economic tool used to verify whether it is worth investing in a given technology based on a cost recovery and potential profit (Masso et al., 2016). The application of P and / or inoculant were profitable for about 64–75% of the farmers in the Northern region. This is comparable to the results of Ronner et al. (2016) who reported that about 60–95% of farmers who used P and / or inoculant (I) in a similar trial in Nigeria achieved economic benefit. Masso et al. (2016) and Banka (2016) reported that the application of P and / or inoculant were financially rewarding for farmers in northern Ghana. In Niger, Bielders and Gérard (2015) reported that 36% of farmers who applied Diammonium phosphate (DAP) and / or urea to their millet had VCR greater than 1. Although, the grain yields were low in the Upper West region, about 14–24 % of the farmers achieved economic benefits. For farmers to adopt either P and / or inoculant, a 100% return to investment (break-even) is often not attractive (Bielders and Gérard, 2015; Ronner et al., 2016). This is the case of SSA smallholder farmers who are generally risk averse (Kisaka-Lwayo et al., 2005) cited by Masso et al. (2016), the return to investments should be at least 200% as indicated by Roy et al. (2006). On the basis of a VCR threshold of 2 or more, 31% of the farmers in Northern region who applied P achieved economic benefit, 53% who applied inoculant achieved economic benefit and 45% who applied P in combination with inoculant achieved economic benefit. However, in the Upper West region, only 2% of the farmers benefited from applying P and inoculant. None benefited economically from combined application of P and inoculant. This was expected due to low yields and the relatively higher prices of the inputs. It was observed that achieving higher economic returns depended on the performance of control plots as previously reported by Buerkert et al. (2001) and Bielders and Gérard (2015).

## 5 Conclusion

Combined application of P and I is an effective means of increasing soybean grain yields on smallholder farms. Addition of *Bradyrhizobium* inoculant to P makes it economically attractive for most farmers. However, wide variability in grain yields might occur due to varying soil and environmental factors. This implies that legume-*Bradyrhizobium* inoculation technologies could be targeted to farmers who would benefit most. The results also confirm the null hypothesis that response to *Bradyrhizobium* inoculation and phosphorus fertilizer is highly variable and economically viable. In this study, two approaches for estimating responsiveness and non-responsiveness have been proposed which could further enhance our understanding in the subject matter.
