# Supplementary material for: On-farm evaluation and determination of sources of variability of soybean response to Bradyrhizobium inoculation and phosphorus fertilizer in northern Ghana
Source: Agric Ecosyst Environ. 2018 Nov 15;267:23–32. doi: 10.1016/j.agee.2018.08.007 (PMC6167739; doi:10.1016/j.agee.2018.08.007)
Supplement: Supplementary file 1 [file mmc1.docx]

**Fig. S1**

Fig. S1. Map showing the study locations with dominant soil types at Northern region

**Fig. S2**

Fig. S2. Maps showing the study locations with dominant soil types at Upper West region

**Fig. S3**

Fig. S3. Rainfall distribution during the cropping season in the Upper West region

**Fig. S4**

Fig. S4. Rainfall distribution during the cropping season in the Northern region
