# Supplementary figures and images for: On-farm evaluation and determination of sources of variability of soybean response to Bradyrhizobium inoculation and phosphorus fertilizer in northern Ghana
Source: Agric Ecosyst Environ. 2018 Nov 15;267:23–32. doi: 10.1016/j.agee.2018.08.007 (PMC6167739; doi:10.1016/j.agee.2018.08.007)

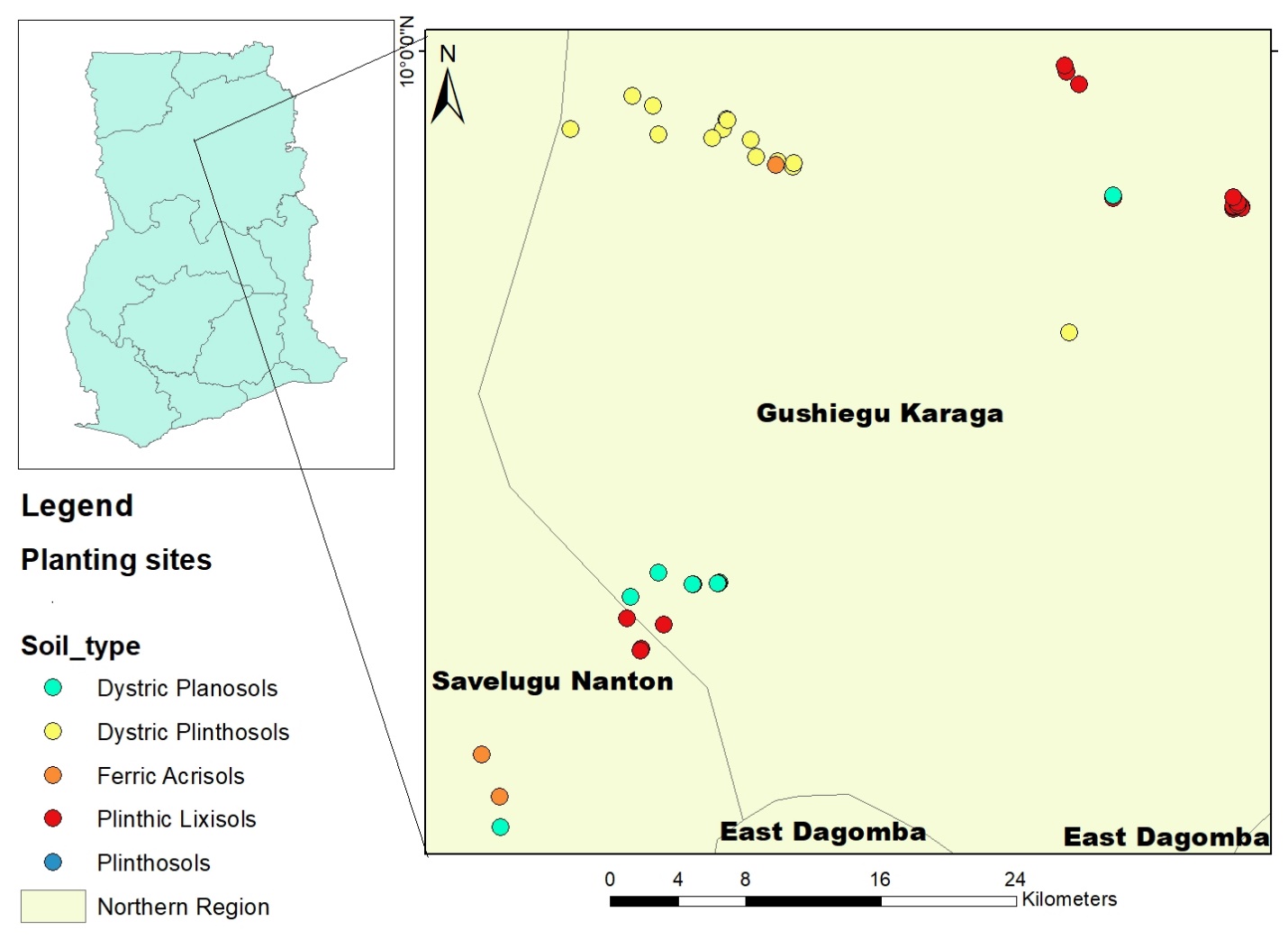

Supplement: Supplementary file 2 [file mmc2.docx]

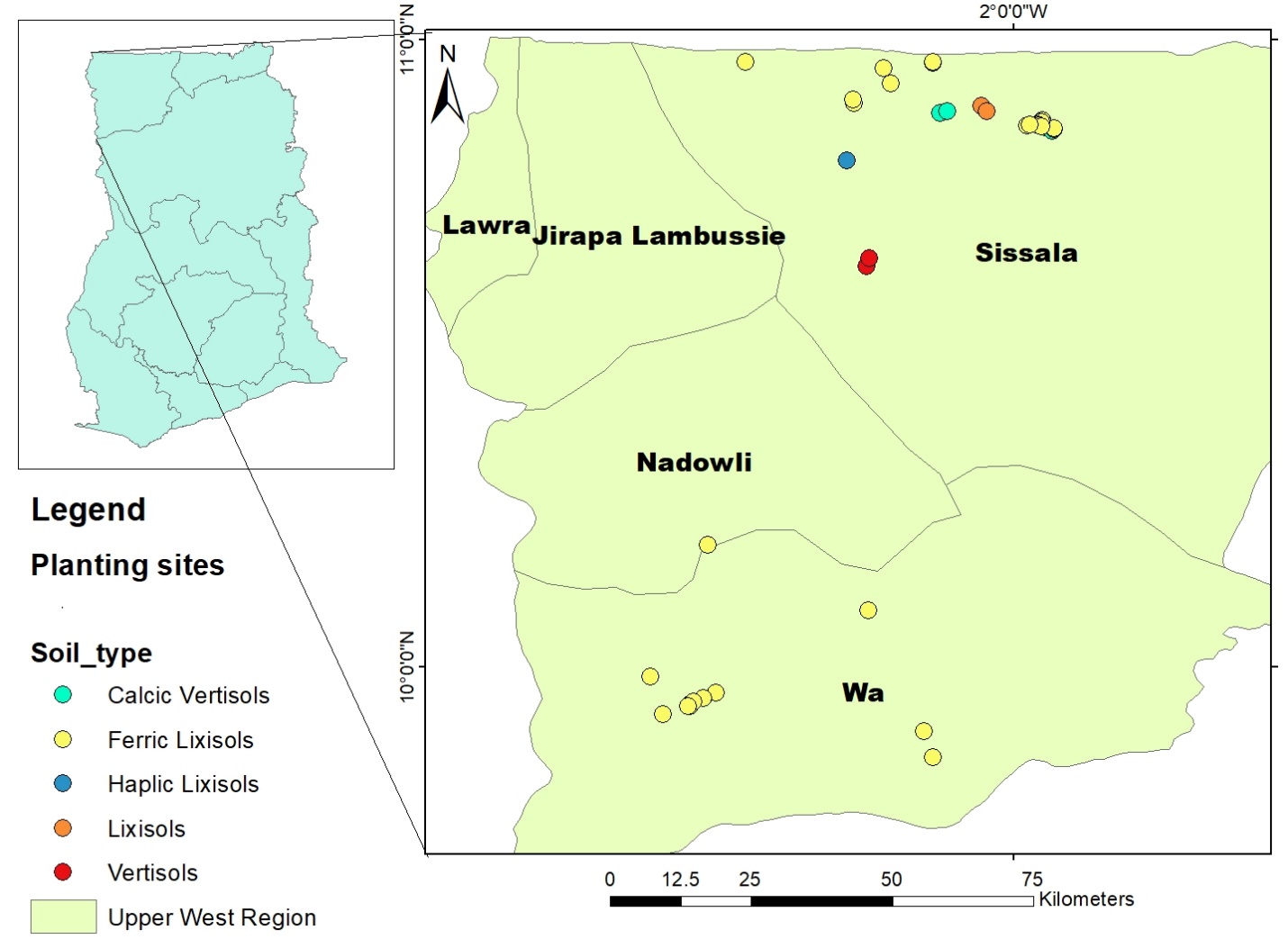

Supplement: Supplementary file 3 [file mmc3.docx]
