# Supplementary material for: On-farm evaluation and determination of sources of variability of soybean response to Bradyrhizobium inoculation and phosphorus fertilizer in northern Ghana
Source: Agric Ecosyst Environ. 2018 Nov 15;267:23–32. doi: 10.1016/j.agee.2018.08.007 (PMC6167739; doi:10.1016/j.agee.2018.08.007)
Supplement: Supplementary file 5 [file mmc5.docx]

Harvesting

Podding

Flowering
