# Supplementary material for: On-farm evaluation and determination of sources of variability of soybean response to Bradyrhizobium inoculation and phosphorus fertilizer in northern Ghana
Source: Agric Ecosyst Environ. 2018 Nov 15;267:23–32. doi: 10.1016/j.agee.2018.08.007 (PMC6167739; doi:10.1016/j.agee.2018.08.007)
Supplement: Supplementary file 4 [file mmc4.docx]

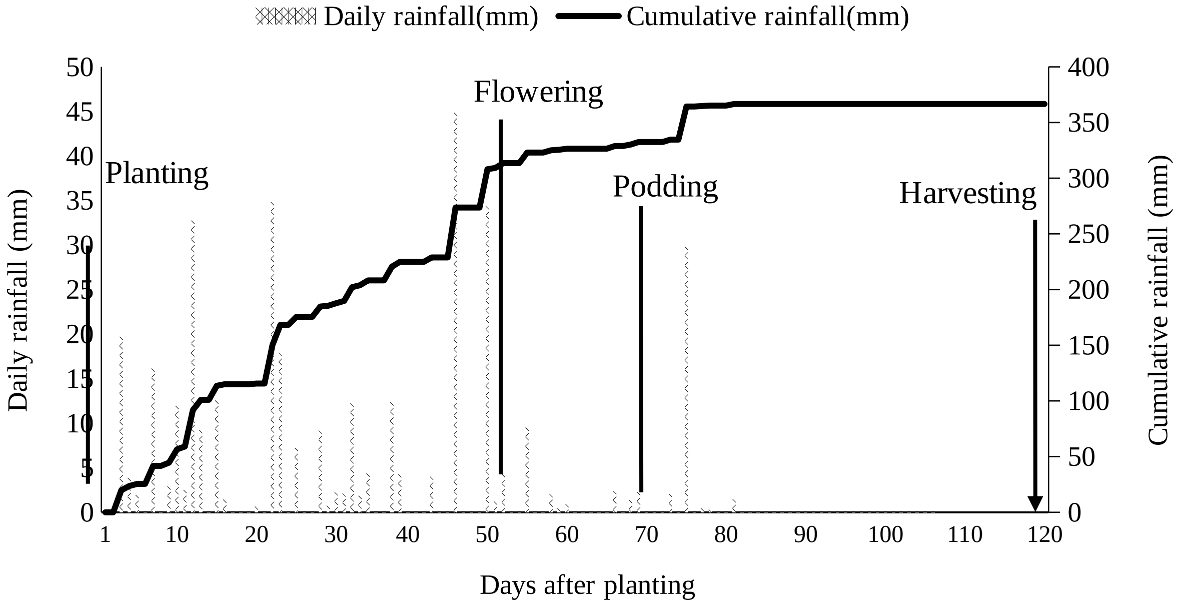

Daily rainfall(mm)
Cumulative rainfall(mm)
Daily rainfall (mm)
Cumulative rainfall (mm)
Planting
Flowering
Podding
Harvesting
Days after planting
0
5
10
15
20
25
30
35
40
45
50
50
100
150
200
250
300
350
400
1
10
20
30
40
50
60
70
80
90
100
110
120
